# Declining Incidence of Candidemia and the Shifting Epidemiology of *Candida* Resistance in Two US Metropolitan Areas, 2008–2013: Results from Population-Based Surveillance

**DOI:** 10.1371/journal.pone.0120452

**Published:** 2015-03-30

**Authors:** Angela Ahlquist Cleveland, Lee H. Harrison, Monica M. Farley, Rosemary Hollick, Betsy Stein, Tom M. Chiller, Shawn R. Lockhart, Benjamin J. Park

**Affiliations:** 1 Mycotic Diseases Branch, Division of Foodborne, Waterborne and Environmental Diseases, Centers for Disease Control and Prevention, Atlanta, Georgia, United States of America; 2 Department of International Health, Johns Hopkins Bloomberg School of Public Health, Baltimore, Maryland, United States of America; 3 Georgia Emerging Infections Program, Atlanta Veterans Affairs Medical Center and Department of Medicine, Emory University School of Medicine, Atlanta, Georgia, United States of America; V.P.Chest Institute, INDIA

## Abstract

**Background:**

Recent reports have demonstrated a decline in bacterial bloodstream infections (BSIs) following adherence to central line insertion practices; however, declines have been less evident for BSIs due to *Candida* species.

**Methods:**

We conducted active, population-based laboratory surveillance for candidemia in metropolitan Atlanta, GA and Baltimore, MD over a 5-year period. We calculated annual candidemia incidence and antifungal drug resistance rates.

**Results:**

We identified 3,848 candidemia cases from 2008–2013. Compared with 2008, candidemia incidence per 100,000 person-years decreased significantly by 2013 in both locations (GA: 14.1 to 9.5, p<0.001; MD: 30.9 to 14.4, p<0.001). A total of 3,255 cases (85%) had a central venous catheter (CVC) in place within 2 days before the BSI culture date. In both locations, the number of CVC-associated cases declined (GA: 473 to 294; MD: 384 to 151). *Candida albicans* (CA, 36%) and *Candida glabrata* (CG, 27%) were the most common species recovered. In both locations, the proportion of cases with fluconazole resistance decreased (GA: 8.0% to 7.1%, −10%; MD: 6.6% to 4.9%, −25%), while the proportion of cases with an isolate resistant to an echinocandin increased (GA: 1.2% to 2.9%, +147%; MD: 2.0% to 3.5%, +77%). Most (74%) echinocandin-resistant isolates were CG; 17 (<1%) isolates were resistant to both drug categories (multidrug resistant [MDR], 16/17 were CG). The proportion of CG cases with MDR *Candida* increased from 1.8% to 2.6%.

**Conclusions:**

We observed a significant decline in the incidence of candidemia over a five-year period, and increases in echinocandin-resistant and MDR *Candida*. Efforts to strengthen infection control practices may be preventing candidemia among high-risk patients. Further surveillance for resistant *Candida* is warranted.

## Introduction

Many healthcare-associated infections (HAIs), including some types of bloodstream infections (BSIs), are a preventable cause of morbidity and mortality in healthcare facilities. In an effort to reduce HAIs, the adoption of new healthcare policies, such as those tied to Medicare reimbursement, have incentivized reducing infection rates and produced mixed results [1–6].

BSIs caused by *Candida* species, also known as candidemia, remain an important public health problem [7–9]. Recent reports have documented significant declines in some bacterial central line associated bloodstream infections (CLABSIs) following adherence to established central line insertion practices, however, similar declines have been less evident for CLABSIs due to *Candida* species [10–14]. *Candida* spp. are common gastrointestinal flora and while many *Candida* BSIs can be attributed to the presence of a central line, some *Candida* spp. are also hypothesized to cause infections by translocation across the gastrointestinal tract unrelated to central line insertion practices [15, 16]. Therefore, line insertion bundles developed to reduce CLABSIs alone may not be completely effective in reducing *Candida* BSIs.

In 2012, we reported a substantial increase over a twenty-year period in the overall population-based incidence of candidemia using population-based surveillance for candidemia in two U.S. locations [9, 17]. This surveillance system captures all culture-confirmed BSIs caused by *Candida* spp., not just healthcare-associated CLABSIs that are tied to mandatory reporting and reimbursement policies, and provide a more complete picture of the burden of candidemia in these communities. The earlier report also noted a marked shift in the species distribution among causative organisms with a significant increase in *Candida glabrata* (CG), a species more likely to be resistant to azoles, the standard antifungal drug of choice.

To monitor more recent changes in the incidence of candidemia and antifungal drug resistance, we now report trends over a five-year surveillance period using population-based prospective surveillance for candidemia in metropolitan Atlanta, Georgia and Baltimore City and County, Maryland.

## Methods

### Surveillance population

Surveillance for candidemia was conducted among residents of Atlanta, Georgia (Fulton, DeKalb, Cobb, Gwinnett, Clayton, Douglas, Newton, and Rockdale counties; 25 hospitals, population: 3.8 million) and Baltimore City and County, Maryland (15 hospitals, population: 1.4 million). Data were collected for five years at each location (March 1, 2008—February 28, 2013 in Atlanta; June 1, 2008—May 31, 2013 in Baltimore).

### Surveillance methods

The methods of surveillance have been previously described [9]. Briefly, a case of candidemia was defined as a blood culture positive for a *Candida* species collected from a resident of the surveillance area at least 30 days apart from any other blood culture positive for *Candida* spp. Cases were classified as: (1) *hospital-onset* (HO) if the first culture was obtained >3 days after admission (with admission being day 1), (2) *healthcare-associated community-onset* (HACO) if the culture was obtained ≤3 days after admission in a patient with recent healthcare exposure, or (3) *community-associated* (CA) if the culture was obtained as an outpatient or ≤3 days of admission in a patient without documentation of recent healthcare exposure. Basic demographic and clinical information was collected on all cases. Surveillance personnel used standardized case report forms to abstract data from medical records. Laboratory records from all participating laboratories were audited monthly.

### Isolate collection, identification, and antifungal susceptibility testing

All available isolates were sent to CDC for species confirmation and antifungal drug susceptibility testing; testing methods have been previously described [18]. CDC-confirmed species are reported; if no isolate was received at CDC, local laboratory species identifications are reported. Because in vitro susceptibility testing may demonstrate susceptibility to one echinocandin but resistance to another that is of questionable clinical correlation [19], molecular testing was used to confirm that the majority of the CG isolates with in vitro echinocandin resistance had a mutation that has been associated with clinical failure [20].

### Statistical methods and denominators

Incidence rates were calculated using year specific population estimates for Baltimore [21] and Atlanta [22] and are presented per 100,000 person-years. Age-adjusted incidence rates did not vary substantially over the five year surveillance period from crude rates, and thus we report crude annual incidence rates of candidemia.

Categorical variables were analyzed using chi-square tests or Fisher’s exact tests. We used a Poisson regression model adjusting for age group to evaluate the change in overall and species specific annual incidence rates over the 5 year surveillance period. In all analyses, the level of significance was set at α = 0.05. All analyses were done using SAS software (version 9.3, SAS Institute, Inc., Cary, NC).

### Human subjects

Patient data were de-identified at each site prior to access and analysis by CDC. CDC conducted ethical review of this surveillance project and deemed it a non-research activity. This activity was also evaluated individually at each location, and was either deemed a public health assessment or human subjects research, and approved by local review boards. In the Baltimore area, the Maryland Department of Health and Mental Hygiene's Institutional Review Board reviewed and approved the protocol, and the protocol was determined exempt from review by the Johns Hopkins Bloomberg School of Public Health Institutional Review Board. In the Atlanta area, the protocol was reviewed and approved by the Emory University Institutional Review Board, and the protocol was determined exempt from review by the Georgia Department of Public Health Review Board.

## Results

### Case-Patient Characteristics

During the 5-year surveillance period, we detected 3,848 cases of candidemia: 2,324 (60%) in the Atlanta area (ATL) and 1,524 (40%) in Baltimore City and County (BTM, Table 1). The median age was 58 years (range, 1 day—101 years), 1,992 (52%) were male, and 2,200 (60%) were black. *Candida albicans* (CA) was the most common species recovered (1,401 cases, 36%), followed by *Candida glabrata* (CG, 27%), *Candida parapsilosis* (CP, 15%), *Candida tropicalis* (CT, 9%), and other species (6%), and 6% of cases had >1 species recovered from their initial blood culture. A total of 225 (7%) cases had an isolate that was resistant to fluconazole including 110/1049 (10%) CG. Fifty-five (2%) cases had an isolate that was resistant to an echinocandin including 43/1049 (4%) CG; 17 (<1%) cases had an isolate that was multidrug resistant (MDR), defined as resistant to fluconazole and one or more echinocandins. Of the MDR cases, 16/17 (94%) were CG, representing 1.5% (16/1049) of CG isolates; 7/17 (44%) MDR cases occurred among persons without documented echinocandin exposure in the previous 14 days.

**Table 1:** Selected characteristics of patients with candidemia in Atlanta and Baltimore metropolitan areas.

		Atlanta		Baltimore		Total	
Characteristic		n	(%)	n	(%)	n	(%)
**Cases**		2324	(60)	1524	(40)	3848	(100)
**Median Age, years (range)**		58	(1 d-101 yrs)	58	(1 d-98 yrs)	58	(1 d-101 yrs)
**Male sex**		1196	(52)	796	(52)	1992	(52)
**Black race**		1427	(61)	873	(57)	2300	(57)
**CVC in place**							
	All ages	1991	(86)	1264	(83)	3255	(85)
	Age <1 year	55	(72)	34	(92)	89	(79)
	Age > = 65	666	(80)	434	(76)	1100	(78)
**Resistance to fluconazole**		136	(07)	89	(06)	225	(07)
**Resistance to an echinocandin**		26	(01)	29	(02)	55	(02)
**Resistance to both flu and echino** *		8	(<1)	9	(<1)	17*	(<1)
**Species**							
	*C*. *albicans*	921	(40)	480	(32)	1401	(36)
	*C*. *glabrata*	589	(25)	460	(30)	1049	(27)
	*C*. *parapsilosis*	390	(17)	204	(13)	594	(15)
	*C*. *tropicalis*	191	(08)	174	(11)	365	(09)
	*C*. *krusei*	38	(02)	15	(01)	53	(01)
	*>1 species*	100	(04)	110	(07)	210	(06)
	*C*. *dubliniensis*	16	(01)	41	(03)	57	(02)
	*Other*	76	(03)	38	(03)	114	(03)
**Age groups, n (incidence rate per 100,000 person years)**							
	<1 year	76	(28.1)	37	(39.5)	113	(33.8)
	1–19 years	88	(1.7)	33	(2.0)	121	(1.9)
	20–44 years	429	(6.1)	298	(11.9)	727	(9.0)
	45–64 years	899	(19.1)	574	(30.0)	1473	(24.6)
	> = 65 years	832	(51.2)	576	(60.1)	1408	(55.7)

*16/17 isolates were *C*. *glabrata* species

### Changes in Incidence Rates

**Fig 1:**
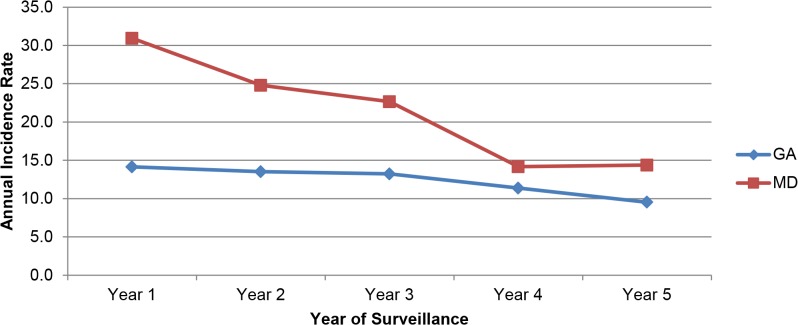
Annual candidemia incidence rates per 100,000 person-years, by year and location, 2008–2013.

Crude annual incidence rates per 100,000 person-years are illustrated in Fig. 1. Compared with 2008 (year 1), the incidence of candidemia decreased significantly in both locations: in ATL, incidence rates declined 33% from 14.1/100,000 from the first year of surveillance to 9.5/100,000 by year 5 (*p*<0.001). In BTM, incidence rates declined 54% from 30.9/100,000 from year 1 to 14.4/100,000 by year 5 (*p*<0.001).

**Fig 2:**
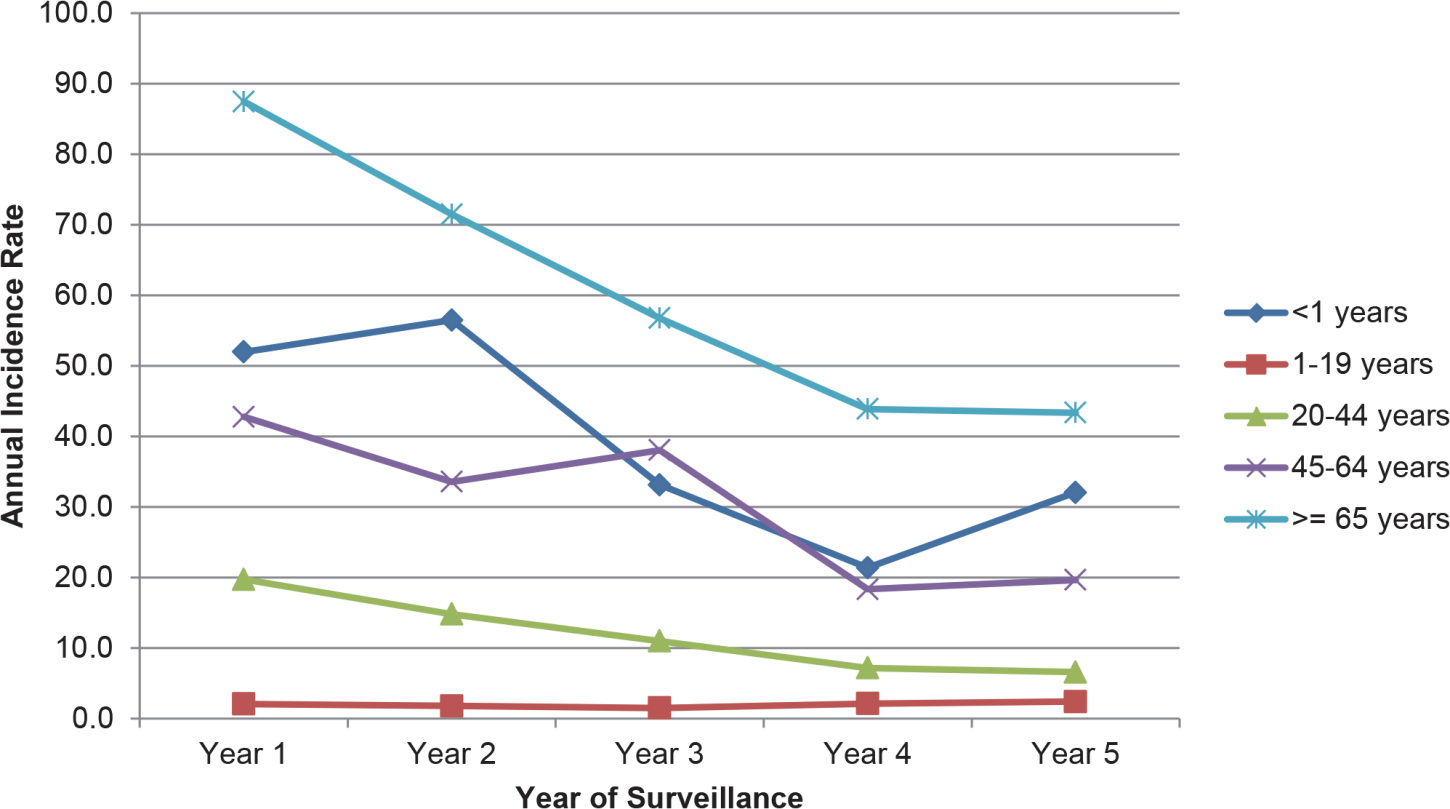
Annual candidemia incidence rates per 100,000 person-years, by year and age-group in the Metropolitan Baltimore area.

**Fig 3:**
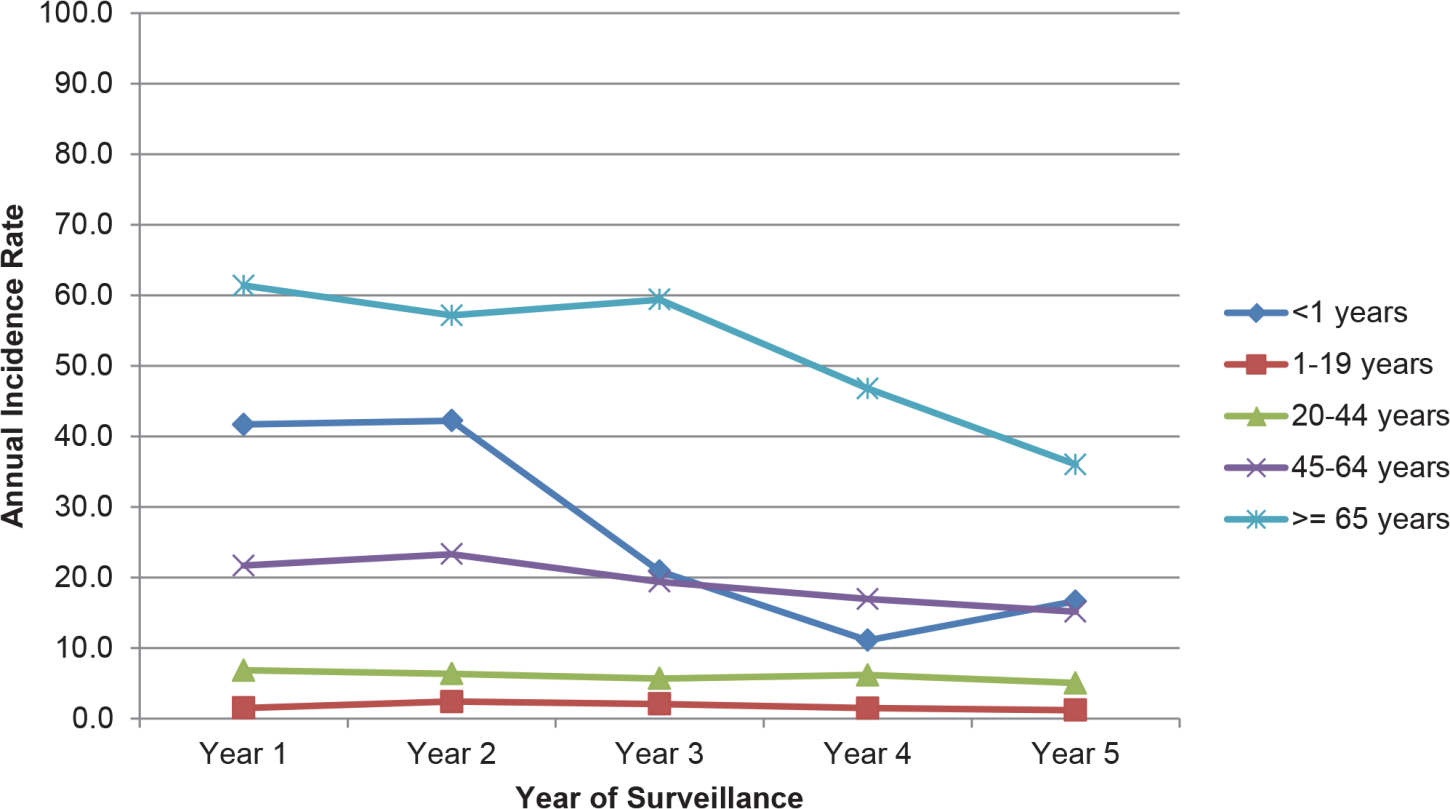
Annual candidemia incidence rates per 100,000 person-years, by year and age-group in the Metropolitan Atlanta area.

Infants aged <1 year and adults aged ≥65 years had the highest rates of candidemia out of all age groups (infants: 33.8/100,000; adults: 55.7/100,000; Table 1). Analysis of age-specific incidence rates per 100,000 person-years demonstrated a decline in every age group except among persons aged 1–19 years in BTM, which increased 17% (from 2.0/100,000 in year 1 to 2.4/100,000 in year 5); all other age groups demonstrated a decline (Fig. 2 and Fig. 3). In ATL, the decline was greatest for persons aged <1 year (from 41.7 to 16.6, −60% decline), followed by persons aged ≥65 years (from 61.3 to 36.0, −41%,) and aged 45–64 years (from 21.7 to 15.1, −30%) (Fig. 2). In BTM, the decline was greatest for persons aged 20–44 years (from 19.7 to 6.6, −67%), followed by persons aged 45–64 years (from 42.7 to 19.6, −54%), ≥65 years (from 87.4 to 43.3, −50%) and <1 year (from 51.9 to 32.0, −38%) (Fig. 3).

### Hospital-specific Declines

**Fig 4:**
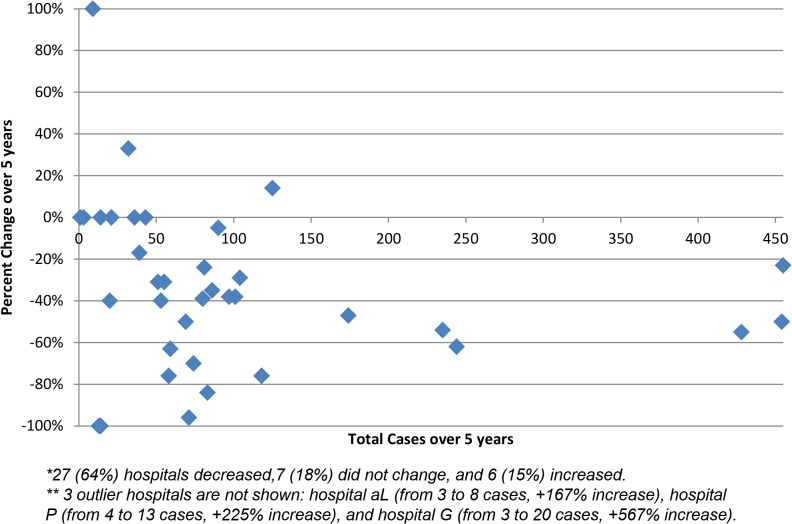
Changes in candidemia case count at participating hospitals over the five-year study period (2008–2013), by the total number of cases each hospital contributed during the study.

Among 40 hospitals under surveillance, 85% reported either a decrease or no change in the frequency of cases over the surveillance period (Fig. 4). Additionally, of the eight hospitals that contributed at least 100 cases during the 5 years of surveillance, there was a substantial drop in the number of cases identified: in ATL 319 cases were identified in the first year, and 190 were identified in the last year (−40%); in BTM, 282 cases were identified in the first year and 125 were identified in the last year (−56%).

### High Risk Groups

In both locations, the frequency of hospital onset (HO) and healthcare-associated community onset (HACO) cases decreased, but community-associated (CA) did not: in ATL, HO cases declined from 405 in year 1 to 226 in year 5 (−44%), HACO declined from 124 to 111 (−10%), and CA changed from 12 to 14 (+17%);. In BTM, HO cases declined from 253 to 111 (−56%), HACO dropped from 175 to 79 (−55%), and CA changed from 12 to 15 (+25%) (Table 2). Among the 135 CA cases, 58 (43%) had diabetes (49% in ATL; 35% in BTM).

**Table 2:** Selected characteristics of candidemia patients by year of surveillance, in Atlanta (ATL) and Baltimore (BTM) metropolitan areas.

Characteristic		Y1	Y2	Y3	Y4	Y5	Change
**Total Cases**							
**ATL**		541	526	487	419	351	−35%
**BTM**		440	354	323	202	205	−53%
**Crude Incidence Rate**							
**ATL**		14.1	13.5	13.2	11.4	9.5	−33%
**BTM**		30.9	24.8	22.7	14.2	14.4	−54%
**Patient group (n)**							
**ATL**	*HO*	405	374	335	270	226	−44%
*HACO*		124	135	136	128	111	−10%
*CA*		12	17	16	21	14	17%
**BTM**	*HO*	253	223	182	121	111	−56%
*HACO*		175	122	131	72	79	−55%
*CA*		12	9	10	9	15	25%
**Central Venous Catheter within 2 days of candidemia (n)**							
**ATL**	*All ages*	473	454	421	349	294	−38%
*<1 year of age*		14	22	6	4	9	−36%
*> = 65 years of age*		159	138	158	118	93	−42%
**BTM**	*All ages*	384	312	262	155	151	−61%
*<1 year of age*		10	9	6	3	6	−40%
*> = 65 years of age*		136	114	77	58	49	−64%
**Surgery among patients who did not have a CVC (n)**							
**ATL**	*Ab surgery*	11	6	6	9	6	−45%
*Non ab surgery*		7	4	6	6	7	0%
**BTM**	*Ab surgery*	6	11	10	6	6	0%
*Non ab surgery*		7	5	3	5	7	0%
**Species-Specific Incidence Rates (IR)**							
**ATL**	*CA*	5.91	5.73	5.59	4.86	3.72	−37%
*CG*		4.28	3.62	3.35	2.72	2.86	−33%
*CP*		2.38	2.62	2.39	2.25	1.63	−31%
*CT*		0.71	0.80	1.22	0.90	0.73	4%
*CK*		0.27	0.26	0.19	0.19	0.25	−8%
*Other*		0.42	0.54	0.70	0.38	0.46	8%
**BTM**	*CA*	11.60	9.18	7.72	4.56	5.12	−56%
*CG*		9.34	8.14	8.20	5.68	4.97	−47%
*CP*		5.41	4.07	3.51	1.69	1.47	−73%
*CT*		3.53	3.03	3.15	1.33	1.97	−44%
*CK*		0.43	0.37	0.14	0.14	0.28	−35%
*Other*		0.93	0.99	1.20	0.47	0.69	−26%
**% Resistant to Fluconazole**							
*ATL*		8%	9.38%	5.67%	7%	7.14%	−10%
*BTM*		6.57%	8.07%	4.73%	4.50%	4.93%	−25%
**% Resistant to an Echinocandin**							
*ATL*		1.18%	1.44%	0.99%	0.83%	2.92%	147%
*BTM*		1.95%	0.58%	2.21%	2.50%	3.45%	77%
**% Multidrug Resistant Candida**							
*ATL*		0.18%	0.38%	0%	0.48%	0.85%	372%
*BTM*		0.91%	0%	0.62%	0.50%	0.98%	8%

***Note*:** *HO*, *healthcare onset*. *HACO*, *healthcare-associated community-onset*. *CA*, *community-acquired*. *Ab surgery*, *abdominal surgery*. *Y*, *year*.

**Fig 5:**
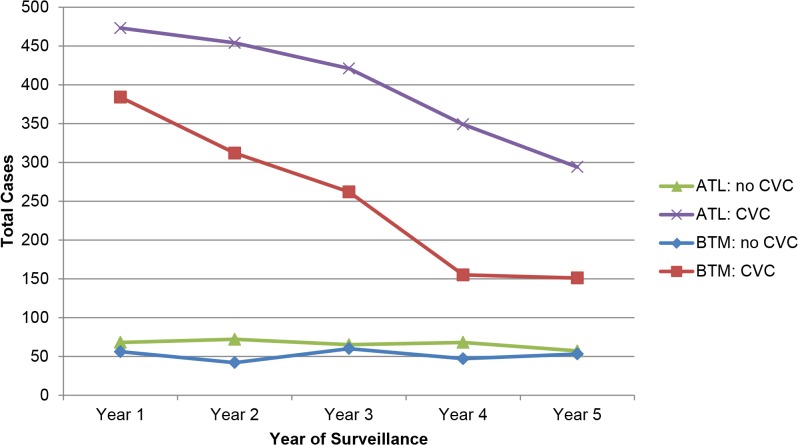
Candidemia cases that had a CVC compared with cases that had no CVC, in Atlanta (ATL) and Baltimore (BTM), 2008–2013.

A total of 3,255 cases (85%) had a central venous catheter (CVC) in place within 2 days prior to the date of their initial culture positive for *Candida* spp. (Table 1). In ATL, CVC-associated cases (n = 1,991) declined from 473 (87%) in year 1 to 294 (84%) in year 5 (−38%) (*p* = 0.030); in BTM, the number of cases with a CVC declined from 384 (87%) to 151 (74%) (−61%) (*p*<0.001) (Fig. 5). The number of non-CVC-associated cases declined in ATL from 68 (13%) to 57 (16%); and in BTM from 56 (13%) to 53 (26%).

### Changes in species distribution

Species-specific incidence rates of all species decreased from year 1 to year 5 in both locations except for CT in ATL, which increased 4% (from 0.71/100,000 to 0.73/100,000, Table 2). In ATL, CA had the largest drop in incidence (from 5.91/100,000 in year 1 to 3.72/100,000 in year 5, −37%), followed by CG (from 4.28 to 2.86, −33%), and CP (from 2.38 to 1.63, −31%). In BTM, CP had the greatest drop in incidence (from 5.41 to 1.47, −73%), followed by CA (from 11.6 to 5.12, −56%), and CG (from 9.34 to 4.97, −47%).

### Change in antifungal drug resistance

In both locations, the overall percent of cases with an isolate resistant to fluconazole decreased (ATL: from 8.0% to 7.1%, −10%; BTM: from 6.6% to 4.9%, −25%), while the overall percent of cases with an isolate resistant to an echinocandin increased (ATL: from 1.2% to 2.9%, +147%; BTM: from 2.0% to 3.5%, +77%; Table 2); 41 (75%) isolates resistant to an echinocandin were CG; other echinocandin-resistant species included CA (n = 7), CT (n = 3), CP (n = 2), and CD (n = 1). The proportion of CG cases due to multi-drug resistant (MDR) *Candida*, increased from 1.8% in 2008 to 2.6% in 2013.

## Discussion

This report from the largest U.S. population-based surveillance program for candidemia to date describes a significant decline in the overall incidence of candidemia in two metropolitan regions. This decline was seen in almost all age groups, was primarily among patients with healthcare exposure, and was especially notable among cases with a central venous catheter. Importantly, we also report an increase in echinocandin resistance as well as the emergence of multi-drug resistant *Candida* BSIs.

In 2012, we reported an increase in candidemia incidence over a twenty year period, as seen through two periodic measurements using population-based surveillance [9], that was attributed to multifactorial changes in patient populations, including possible increases in patient populations at high risk for candidemia.

In comparison, our current report documents a subsequent decrease in incidence that is not likely to be due to changes in high-risk populations; rather, it is probable that the declines noted here are related to healthcare delivery improvements. We found that declines were predominantly among candidemia cases with healthcare exposure, but not among the small number of community-associated cases, suggesting that factors associated with healthcare delivery may be driving this decline. Our data further demonstrate declines among patients with *Candida* BSIs occurring in the presence of CVCs, while the number of cases without CVCs did not decline as substantially. Taken together, these data suggest that the large declines noted here may be the result of policies and practices related to catheter insertion and maintenance.

Recent policies targeted at reducing healthcare-associated infections, including state mandates for public reporting, have incentivized reducing CLABSIs [1]. Subsequent reports including data from CDC’s National Health Safety Network (NHSN) have described large declines in CLABSI rates [13, 23, 24]. Data from our active surveillance system, which is not tied to any reimbursement or reporting mechanism, support the trends observed through systems such as NHSN and suggest these declines are robust. Anecdotally, most hospitals in our surveillance area have recently introduced policies that mandate improvements in catheter care (data not shown); further study is needed to understand if declines can be attributed to these policies.

Notably, while we documented a drop in fluconazole resistance, we also report a small but concerning increase in isolates resistant to echinocandins, and the emergence of multidrug resistant (MDR) *Candida*, almost all of which were the species CG. Although the decrease in fluconazole resistance is reassuring, it may be partially due to the increased use of echinocandins as primary therapy for candidemia. This shift in practice, particularly for CG infections, may be contributing to the increase in echinocandin resistance; recent reports have demonstrated the emergence of echinocandin resistant CG[17, 25, 26], possibly driven by wider usage of this drug class as primary therapy[27] and prophylaxis. Although the modes of action and target sites of the triazoles (i.e. fluconazole) and echinocandins are different[28], CG are the most common fluconazole-resistant species, and mutations conferring echinocandin resistance in CG can emerge quickly[29]. Microbiology laboratories should consider antifungal susceptibility testing for CG isolates, since detection of echinocandin-resistant or MDR *Candida* can influence therapy.

It is also concerning that 44% of MDR *Candida* occurred in patients without recent exposure to echinocandins. This observation suggests person-to-person transmission of this resistant phenotype is possible, as has been previously suggested [30]. Vigilant monitoring for resistance among CG species will continue to be critical, and further investigation into the possible transmission of MDR *Candida* will be essential for targeting prevention efforts.

This report is subject to several limitations. Although we suspect that the dramatic declines in candidemia occurring in patients with a CVC were due to improved catheter care, it is possible that declines were due to some external factor such as reductions in overall CVC usage in a particular ward. We report large declines in most hospitals under surveillance, but do not collect data on number of hospital admissions or other hospital denominator data, and are therefore unable to report if the declines are a true decrease in the risk of disease or if this is related to a shift in healthcare utilization (e.g., change in central line utilization, referral patterns, frequency of blood culture collection, etc). However, the majority of hospitals in our surveillance system demonstrated declines indicating a trend not isolated to a few institutions.

This report describes significant declines in the incidence of candidemia bloodstream infections in two major U.S. locations over a 5 year period, and an increase in echinocandin resistant CG. Continued surveillance will be important to help understand factors contributing to these declines and to monitor for the emergence of resistant *Candida*.
